# Adaptation of Nontypeable *Haemophilus influenzae* in Human Airways in COPD: Genome Rearrangements and Modulation of Expression of HMW1 and HMW2

**DOI:** 10.1128/mbio.00140-23

**Published:** 2023-03-16

**Authors:** Timothy F. Murphy, Charmaine Kirkham, Adonis D’Mello, Sanjay Sethi, Melinda M. Pettigrew, Hervé Tettelin

**Affiliations:** a Division of Infectious Diseases, Department of Medicine, University at Buffalo, The State University of New York, Buffalo, New York, USA; b Department of Microbiology and Immunology, Institute for Genome Sciences, University of Maryland School of Medicine, Baltimore, Maryland, USA; c Division of Pulmonary, Critical Care and Sleep Medicine, Department of Medicine, University at Buffalo, The State University of New York, Buffalo, New York, USA; d Department of Medicine, Veterans Affairs Western New York Healthcare System, Buffalo, New York, USA; e Department of Epidemiology of Microbial Diseases, Yale School of Public Health, New Haven, Connecticut, USA; The University of Mississippi Medical Center

**Keywords:** *Haemophilus influenzae*, genome analysis, pathogenesis, persistence, respiratory pathogens

## Abstract

Chronic obstructive pulmonary disease (COPD) is a common debilitating disorder that is the third most common cause of death globally. Chronic lower airway infection by nontypeable Haemophilus influenzae (NTHi) in adults with COPD increases airway inflammation, causes increased symptoms, and accelerates progressive loss of lung function. Little is known about the mechanisms by which NTHi survives in COPD airways. To explore this question, the present study analyzes, in detail, 14 prospectively collected, serial isolates of a strain that persisted for 543 days in a patient with COPD, including analysis of four gap-free complete genomes. The NTHi genome underwent inversion of a ~400-kb segment three times during persistence. This inversion event resulted in switching of expression of the HMW1A and HMW2A adhesins as the inversion sites are in the promoter regions of HMW1 and HMW2. Regulation of the level of expression of HMW 1 and HMW2 in the human airways was controlled by the ~400-kb inversion and by 7-bp repeats in the HMW promoters. Analysis of knockout mutants of the persistent strain demonstrated that HMW1 and HMW2 proteins both function in the adherence of NTHi to human respiratory epithelial cells during persistence and that HMW1 also facilitates invasion of epithelial cells. An inverse relationship between biofilm formation and HMW1 expression was observed during persistence. This work advances understanding of the mechanisms of persistence of NTHi in COPD airways, which can inform the development of novel interventions to treat and prevent chronic NTHi infection in COPD.

## INTRODUCTION

Chronic obstructive pulmonary disease (COPD) is a chronic, debilitating disorder that is the third most common cause of death globally (1, 2). Its prevalence is projected to increase in the coming decades because of aging of the population and continued exposure to risk factors, including tobacco use, biomass pollution, and smoke (1, 3). The course of COPD is characterized by intermittent worsening of the disease, called exacerbations, which cause enormous morbidity, including lost work time, clinic visits, emergency room visits, hospital admissions, respiratory failure, and sometimes death (4, 5). Approximately half of COPD exacerbations are caused by bacterial infections, and nontypeable Haemophilus influenzae (NTHi) is the most common bacterial cause. (6, 7).

A second important role of bacteria in the course and pathogenesis of COPD is chronic infection of the lower airways. In addition to causing exacerbations, NTHi persists in the airways of adults with COPD during clinically stable periods (8–11). This chronic infection increases the airway inflammation that is a hallmark of COPD, leads to the progressive loss of lung function, and is associated with increased symptoms (9, 10, 12–14). NTHi is an exclusively human pathogen which has developed mechanisms to adapt and survive in the hostile environment of the human airway. Analysis of genomes of the first and last isolates of 101 NTHi strains that persisted in the airways of adults with COPD demonstrated that NTHi alters its genome in several ways, including genome rearrangement in the form of a large inversion, single nucleotide polymorphisms (SNPs), and simple sequence repeats (SSRs) which mediate slipped-strand mispairing to regulate critical virulence functions to facilitate survival and mediate pathogenesis in the airways of adults with COPD (15).

In the present study, to further explore how NTHi persists in COPD airways, we performed detailed analyses of 14 serial isolates of a strain that persisted for 543 days in a patient with COPD. Our analyses focused on changes in the genomes of serial isolates, changes in the expression of selected genes, and virulence-associated phenotypes. Studying serial isolates will reveal the dynamics of the changes that occur during persistence in human airways, whether these changes are maintained over time, and perhaps other novel observations. Our analyses revealed that (i) the NTHi genome underwent inversion of a ~400-kb segment three times during persistence over 543 days; (ii) the inversion sites were located in the promoter regions of HMW1 and HMW2 adhesins; (iii) the switching of HMW1A and HMW2A expression and modulation of the expression levels of HMW 1A and HMW2A were controlled by the ~400-kb inversion and the number of 7-bp repeats in the *hmw* promoters; (iv) HMW1A and HMW2A proteins both function in the adherence of NTHi to human respiratory epithelial cells during persistence, with HMW1 also facilitating invasion of respiratory epithelial cells; and (v) an inverse relationship exists between expression of HMW1 and biofilm formation. This work serves as a guide to future studies related to the persistence of this exclusively human pathogen which is the most frequent bacterial pathogen in COPD. Elucidating the genomic and phenotypic changes that NTHi undergoes during persistence in the human airway provides a better understanding of mechanisms of persistence and their relative importance in addition to revealing targets for the development of new therapies and vaccines.

## RESULTS

**FIG 1:**
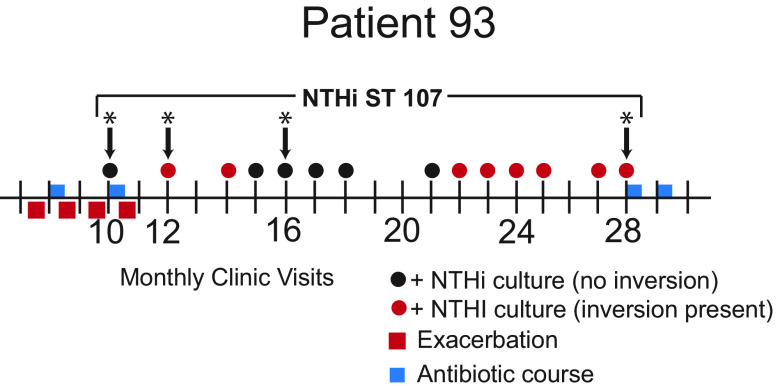
Timeline of patient 93, who was followed prospectively for 1,210 days and 41 monthly clinic visits with sputum cultures. Vertical lines indicate clinic visits. Circles indicate the presence of nontypeable Haemophilus influenzae (NTHi) in sputum culture (black circles: no inversion; red circles: ~400-kb inversion present). Arrows with asterisks (*) indicate gap-free genomes. Red squares indicate clinical exacerbations. Blue rectangles indicate courses of antibiotics. Bracket above the timeline indicates that the patient carried a strain of multilocus sequence type (MLST) 107 from visit 10 through visit 28 (543 days).

In the present study, we report the detailed analysis of 14 serial isolates of a strain of NTHi (multilocus sequence type [MLST] 107) (15) that persisted for 534 days in the airways of patient 93, a 75-year-old adult with COPD who was followed in the study for a total of 1,210 days and 41 clinic visits (Fig. 1). This patient had a 125-pack-year history of smoking and a 13-year history of COPD. He quit smoking 8 years prior to enrolling in the study. His comorbid conditions included coronary artery disease and benign prostatic hypertrophy. Pulmonary function tests showed moderate airway obstruction (forced expiratory volume in 1 s: forced vital capacity = 0.67).

### A ~400-kb segment underwent inversions during persistence.

**FIG 2:**
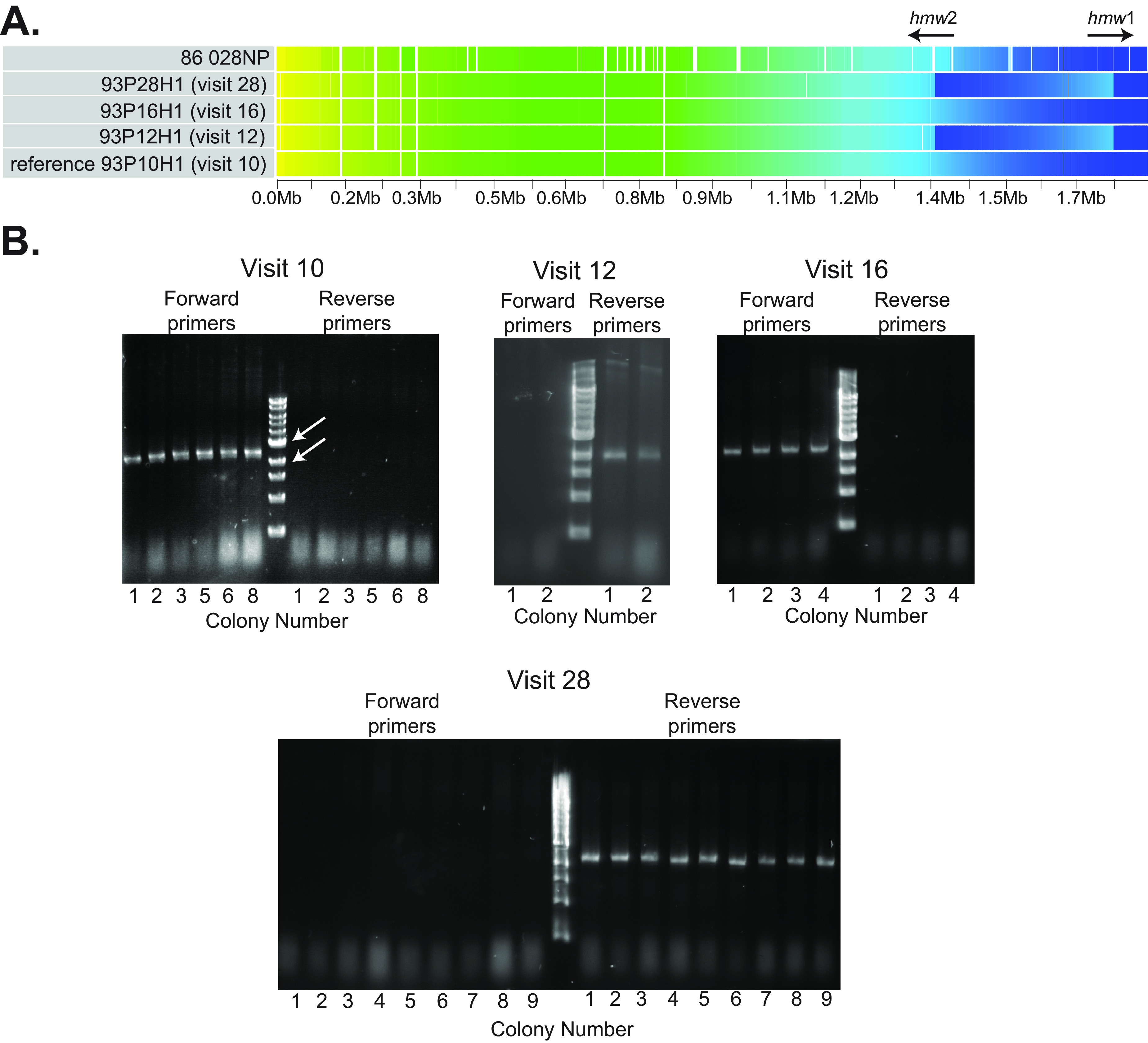
(A) Sybil synteny gradient image of gap-free genomes of NTHi 86 028NP (18) and four isolates of the NTHi strain under study that persisted in chronic obstructive pulmonary disease (COPD) airways for 534 days. Clusters of syntenic orthologous genes were used to draw orthologs of genes predicted in the reference genome (93P10H1). Each ortholog is drawn with the color from the gradient corresponding to its position in its own genome. Breaks in the color gradient in the visit 12 and visit 28 isolates represent inversions in these isolates. White spaces indicate deletions or regions harboring genes that are not included with reference genes in clusters of orthologs. Arrows indicate the location and orientation of the *hmw1* and *hmw2* operons. (B) Ethidium bromide-stained agarose gels. Genomic DNA was purified from cultures grown from individual colonies on original plates from sputum samples obtained from clinic visits of the four isolates with gap-free genome sequences, as noted in each panel. Primers upstream of the 5′ end of the ~400-kb inversion in strain 93P10H1 (visit 10 isolate) (forward primers) and primers downstream of the 3′ end of the inversion (reverse primers) were used to amplify DNA from cultures grown from original colonies from sputum cultures of the four visits as noted. Fragment sizes are between the 2-kb and 3-kb molecular size markers. Results show that all colonies isolated from the original cultures from visits 10 and 16 had the inversion in the forward direction, whereas all colonies from the original cultures from visits 12 and 28 had the inversion in the reverse direction.

Genome analysis of the persistent strain included gap-free (closed or finished) genomes of the first isolate following acquisition of the strain at the time of an exacerbation (93P10H1), the last isolate before the strain was cleared (93P28H1), and two intervening isolates (93P12H1 and 93P16H1) (16, 17). For isolate nomenclature, the first number is the patient number and the number after ‘P’ is the monthly clinic visit number. Synteny gradients illustrate that a ~400 kb segment located toward the 3′ end of the genome underwent inversion three times over 543 days in the patient’s respiratory tract. (Fig. 2A).

To determine whether the segment of the genome in the patient 93 strain that underwent inversion was in the same orientation in all NTHi strains isolated from an individual sputum sample, we studied cultures from individual colonies that grew from sputum on the original culture plates. Up to 10 colonies were individually subcultured and cryopreserved when sputum was processed. The cultures from each of the original colonies of the four isolates of the persistent strain were subjected to PCR to assess the orientation of the inversion. For each of the four isolates for which we have complete gap-free genomes, all colonies from the original culture had the same orientation as the other colonies at that visit (visit 10, six colonies; visit 12, two colonies; visit 16, four colonies; and visit 28, nine colonies) (Fig. 2B).

**FIG 3:**
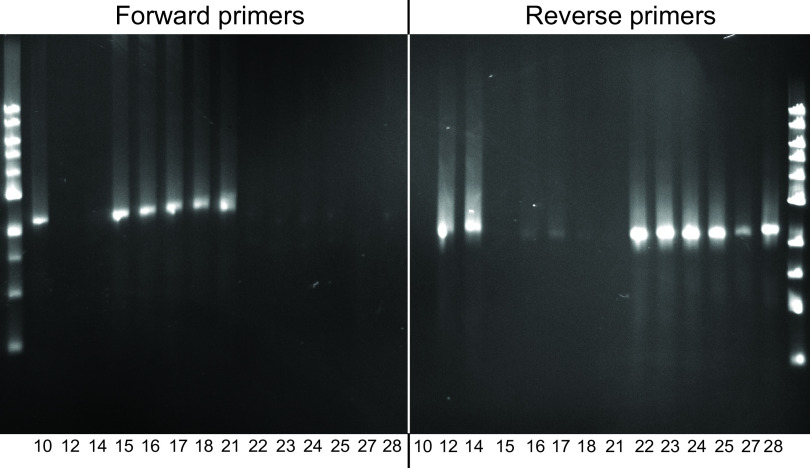
Ethidium bromide-stained agarose gels. Genomic DNA was purified from cultures obtained from clinic visits as noted in each panel. Primers bracketing the 5′ end of the ~400-kb segment in strain 93P10H1 (visit 10 isolate) (forward primers) and primers bracketing the 3′ end of the ~400-kb segment (reverse primers) were used for PCR with genomic DNA. Fragment sizes are between the 2-kb and 3-kb molecular size markers. Results show the direction of the 400-kb genome segment that undergoes inversion in the airways for each of the 14 isolates recorded at monthly clinic visits.

These same PCR primers were used to assess the orientation of the inversion in the intervening isolates and to determine at which point during persistence the inversion occurred. The ~400-kb segment was in the forward configuration (same as the NTHi 86-028NP genome) (18) at visit 10 (~22 days) and visits 15 through 21 (~231 days) and in the reverse configuration at visits 12 through 14 (~81 days) and visits 22 through 28 (~209 days) (Fig. 3). Based on analysis of intervening isolates, we conclude that the NTHi genome undergoes frequent inversion of the ~400-kb segment in its genome during persistence in the airways of adults with COPD. Furthermore, based on analysis of multiple colonies isolated from the original sputum culture, when an inversion occurs, it is present in the entire population of NTHi from an individual sputum sample.

### Expression of HMW1A and HMW2A surface proteins during persistence.

The ~400-kb inversion occurs within the promoter region upstream of the *hmw1* and *hmw2* operons. Each *hmw* operon consists of three genes. The operon that encodes the HMW1 adhesin consists of *hmw1A*, which encodes the adhesin protein HMW1A; *hmw1B*, which encodes a pore-forming translocator protein; and *hmw1C*, which encodes a glycosyltransferase that glycosylates the HMW1A adhesin protein. The operon that encodes the HMW2 adhesin also has three genes, including genes that encode the HMW2A adhesin protein (*hmw2A*), a pore-forming translocator protein (*hmw2B*), and a glycotransferase (*hmw2C*) (19). The HMW1A and 2A adhesin proteins are ~120 kDa and share ~80% amino acid identity, whereas the translocator proteins and glycosyl transferases are 97% to 99% identical (19–22). HMW1A and HMW2A have differing affinities to host cell glycans that may affect adherence properties and cell tropism (21). Given the location of the inversion site in the *hmw1* and *hmw2* operons, we assessed the expression of HMW adhesins during persistence, in each of the 14 isolates, including all intervening isolates, using immunoblot assays with guinea pig antiserum that recognizes both HMW1A and HMW2A (kindly provided by Joseph St. Geme).

**FIG 4:**
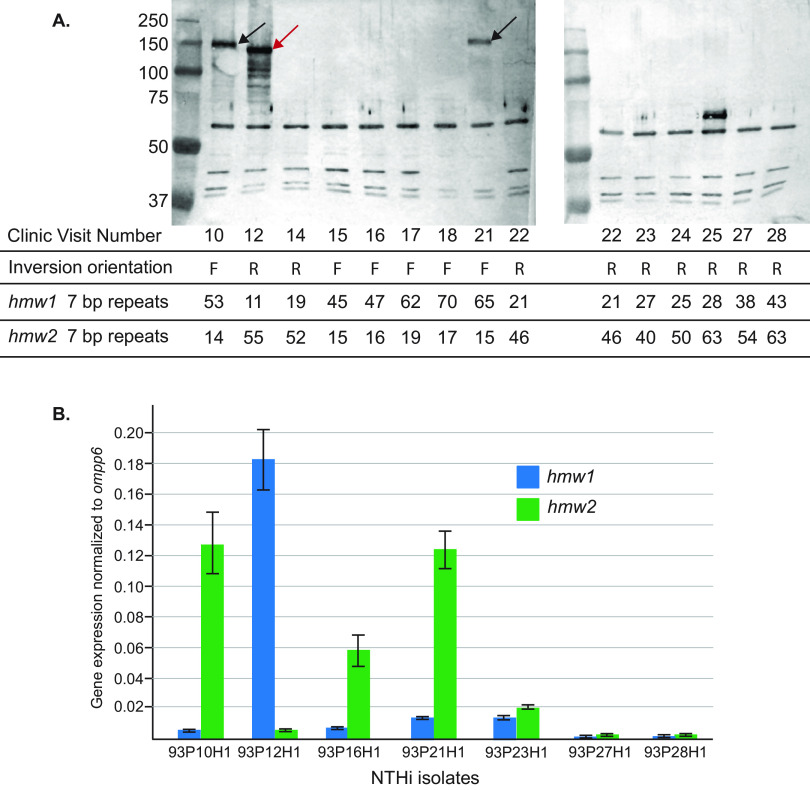
(A) Immunoblot assay of whole-bacterial cell lysates of NTHi isolates from each clinic visit of patient 93 as noted across the bottom. Isolates from each visit which had a positive NTHi culture are shown. The blot was probed with guinea pig antiserum to HMW1A, which recognizes both HMW1A and HMW2A. Black arrows denote HMW2A (visits 10 and 21), red arrow denotes HMW1A (visit 12). Molecular mass markers are noted in kDa on the left. Rows below the blot indicate the orientation of the ~400-kb inversion (F, forward; R, reverse) and the number of 7-bp repeats in the promoter of *hmw* genes of each isolate. (B) Results of quantitative reverse transcriptase PCR (qRT PCR) of *hmw1* and *hmw2* in selected sequential isolates of the patient 93 persistent NTHi strain, including four isolates for which gap-free genome sequences are available in addition to selected isolates between visits 16 and 28 (392 days). *y* axis indicates gene expression normalized to *ompp6.* Values denoted by bars for all assays represent the mean of three independent experiments. Error bars represent standard error of the means.

The expression of HMWs undergoes changes throughout persistence in the respiratory tract. The strain expressed HMW2A upon acquisition by the patient (visit 10). Within 22 days following strain acquisition, a genome inversion occurred, HMW2A was no longer expressed and HMW1A was now expressed (visit 12) (Fig. 4A). The antiserum used detects both HMW1A and HMW2A. The expression of the two proteins at visits 10 and 12 were discriminated from each another by the location of the band on the immunoblot, in which HMW2A migrates at a slightly higher molecular mass in the strain from patient 93. Isolates from subsequent visits showed no expression of either HMW with the exception of visit 21, which showed expression of HMW2A based on the size of the band on the immunoblot.

To further assess expression of *hmw1A* and *hmw2A* in the patient 93 persistent strain, we performed quantitative reverse transcriptase PCR (qRT-PCR) on selected isolates of the strain (Fig. 4B and Table S1 in the supplemental material) using HMW1A-specific and HMW2A-specific primers. The results showed that transcription of isolates at visits 10, 12, 16, 21, 23, 27, and 28 closely paralleled protein expression detected by immunoblot assays with antiserum to HMW1 and HMW2 (Fig. 4).

10.1128/mbio.00140-23.1 TABLE S1 Results of quantitative reverse transcriptase PCR of patient 93 isolates grown *in vitro*. Download Table S1, DOCX file, 0.01 MB. Copyright © 2023 Murphy et al. 2023 Murphy et al. https://creativecommons.org/licenses/by/4.0/ This content is distributed under the terms of the Creative Commons Attribution 4.0 International license.

### Relationship of HMW expression and genome inversion.

HMW1A and 2A undergo phase variation controlled by 7-bp repeats in the promoter region upstream of the structural *hmw1A* and *hmw2A* genes (23, 24). Given that the ~400-kb inversion occurs at the site of the HMW operons, we assessed the relationships of HMW expression with inversions and 7-bp upstream repeats. As the number of 7-bp repeats upstream of *hmw1A* and *hmw2A* increases, the expression of HMW1A and -2A generally decreases, as previously described (23), with some variability in this relationship (Table S2). Note that the isolate at visit 16 shows weak expression in immunoblot assays, depending on the conditions of the assay (data not shown). Interestingly, the inversions put the *hmw* operons under the control of different *hmw* promoters, which harbor different numbers of 7-bp repeats. The isolate at visit 10 (93P10H1) has 53 repeats upstream of *hmw1A* and 14 repeats upstream of *hmw2A* (Table 1), resulting in high-level expression of HMW2A and no expression of HMW1A at visit 10. The ~400-kb inversion then occurs between visits 10 and 12. The isolate at visit 12 (93P12H1) now has 10 repeats upstream of *hmw1A* and 55 repeats upstream of *hmw2A*, resulting in reversed expression of HMWs with high-level expression of HMW1A and no expression of HMW2A (Table 1 and Fig. 4). We conclude that the ~400-kb inversions contribute to the regulation of HMW expression by abruptly altering the number of 7-bp repeats in the promoter regions of the *hmw* operons.

**TABLE 1:** Expression of HMW proteins of selected isolates of patient 93 nontypeable Haemophilus influenzae persistent strains related to the configuration of the ~400-kb inversion

Strain isolate^*a*^	Inversion configuration	HMW1A, located 3′ of inversion		HMW2A, located 5′ of inversion	
		SSRs^*b*^	Expression^*c*^	SSRs	Expression^*c*^
93P10H1	Forward	53	−	14	++
93P12H1	Reverse	10	++	55	−
93P16H1	Forward	47	−	16	+
93P28H1	Reverse	43	−	63	−

a Isolates for which gap-free genome sequences were determined.

b SSRs: number of 7-bp simple sequence repeats in the promoter region.

c HMW expression based on immunoblot assay with antiserum to HMWs. ++, high level expression; +, moderate level expression.

10.1128/mbio.00140-23.2 TABLE S2 Number of repeats in the *hmw* promoters in isolates grown from individual colonies from the original sputum culture plates of the four isolates for which gap-free genomic sequences are available. Download Table S2, DOCX file, 0.02 MB. Copyright © 2023 Murphy et al. 2023 Murphy et al. https://creativecommons.org/licenses/by/4.0/ This content is distributed under the terms of the Creative Commons Attribution 4.0 International license.

To further assess the observation that the 7-bp repeats and inversions contributed to regulation of the expression of HMWs, the segments of the *hmw1* and *hmw2* promoter regions from each individual colony that grew from sputum on the original culture plates were amplified by PCR and the number of 7-bp repeats was determined (Table S2). The number of repeats in each of the 21 total colonies among the four isolates paralleled all colonies in that sputum sample. Isolates that did not express an HMW had a larger number of 7-bp repeats in the promoter (range: 15 to 60), whereas isolates that expressed an HMW had a lower number of 7-bp repeats (11 to 14).

Collectively, these results support the conclusion that HMW1 and HMW2 expression are regulated both by upstream repeats and by an ~400-kb inversion that occurs in the promoter regions of the *hmw1* and *hmw2* operons. Moreover, these results are consistent with the conclusion that the environmental conditions in the human airway at particular times places selective pressure on the NTHi population in the airways to either express or not express an HMW.

### Modulation of adherence and invasion of human respiratory epithelial cells during persistence.

**FIG 5:**
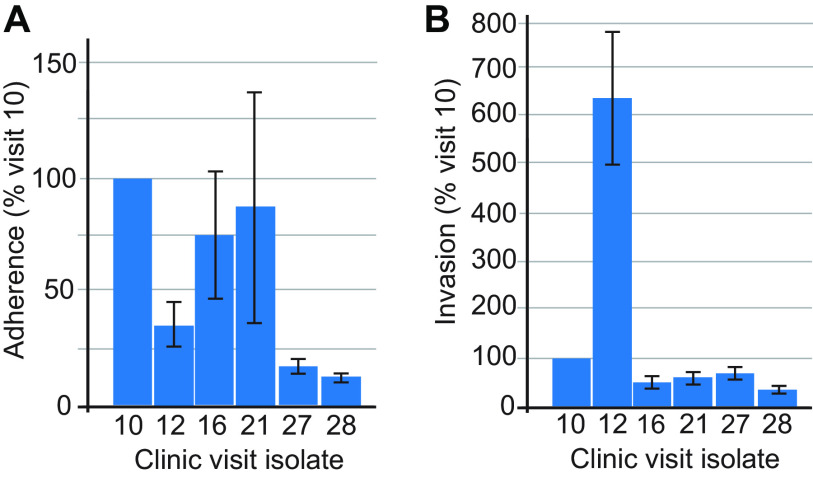
(A) Results of assays showing adherence of selected sequential isolates of patient 93 isolates to H292 human respiratory epithelial cells. (B) Results of assays showing invasion of isolates of the persistent strain. In panels A and B, *x* axes show the clinic visit number of sequential isolates, including the four isolates for which gap-free genome sequences are available in addition to selected isolates between visits 16 and 28 (392 days). *y* axes show results expressed as a percentage of the visit 10 isolate calculated from CFU of NTHi that adhered to or invaded epithelial cells. Values denoted by bars for all assays represent the mean of three independent experiments. Error bars represent standard deviations.

Given the regulation of expression of HMW1A and HMW2A during persistence in the airways of adults with COPD and the function of HMWs in adherence to human respiratory cells, we assessed adherence to and invasion of the H292 human respiratory epithelial cell line by selected sequential isolates of the persistent strain. Figure 5A shows that isolates at visits 10, 16, and 21 express HMW2A and show adherence to epithelial cells. Isolates from visits 12, 27, and 28 do not express HMW2A and show low adherence. By contrast, 93P12H1, the isolate at visit 12, is the only isolate with high-level expression of HMW1A and demonstrates a lower level of adherence than isolates that express HMW2A and a marked increase in epithelial cell invasion, equivalent to 600% greater than that of the isolate at the time of acquisition at visit 10 (Fig. 5B). These results suggest that HMW2A is important for adherence of NTHi during persistence in human COPD airways and that HMW1A contributes to both adherence and invasion of respiratory epithelial cells. These conclusions are confounded by the fact that changes in genomes and expression patterns other than HMWs during persistence contribute to adherence and invasion. Therefore, we engineered HMW knockout mutants to more rigorously assess the role of HMWs in these phenotypes.

**FIG 6:**
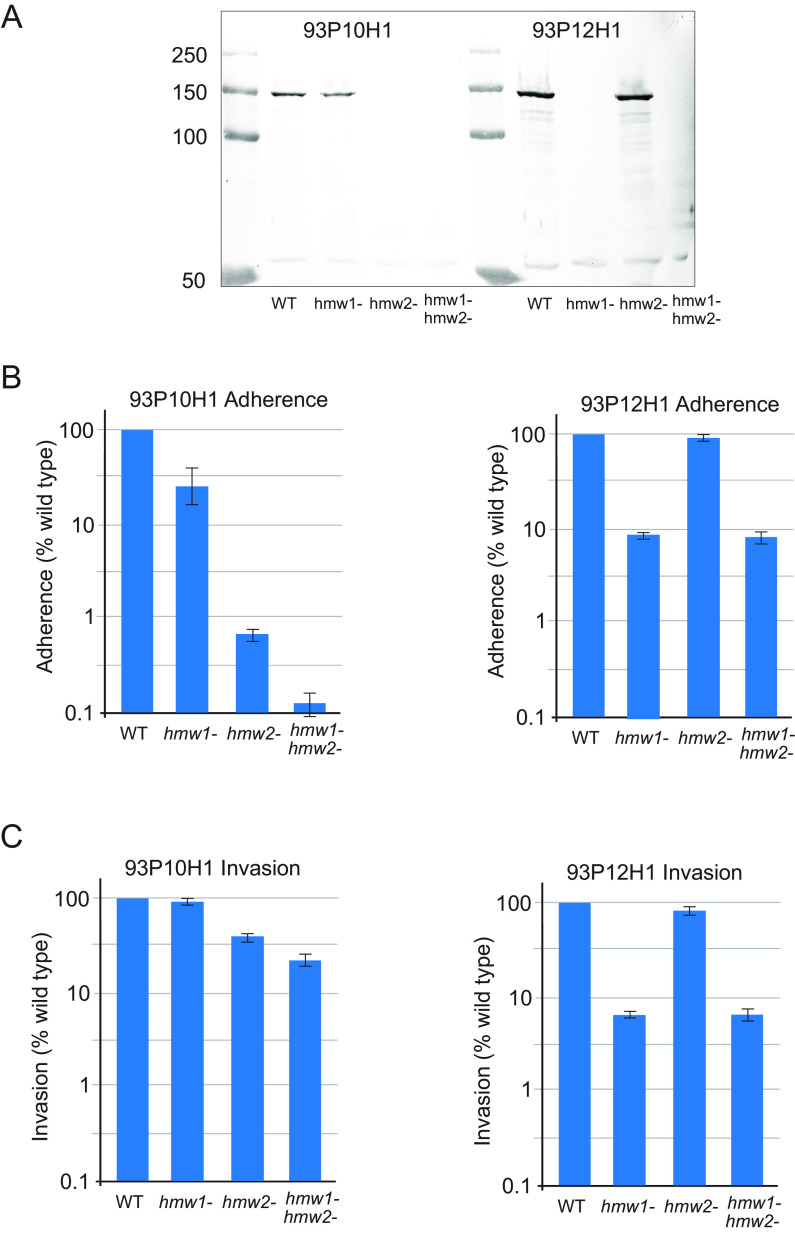
(A) Immunoblot assay of whole-bacterial cell lysates of NTHi isolates 93P10H1 and 93P12H1 and corresponding knockout mutants (*hmw1* knockouts, *hmw2* knockouts, and *hmw1/hmw2* double knockouts). The blot was probed with guinea pig antiserum to HMW1, which recognizes both HMW1 and HMW2. Molecular size markers are noted on the left in kilodaltons. (B) Results of adherence assays of 93P10H1 and 93P12H1 and their corresponding knockout mutants as noted with H292 human respiratory epithelial cells. (C) Results of invasion assays of 93P10H1 and 93P12H1 and their corresponding knockout mutants. In panels B and C, *y* axes show results expressed as a percentage of the visit 10 or 12 isolate calculated from CFU of NTHi that adhered to or invaded epithelial cells. Values for all assays represent the mean of three independent experiments. Error bars represent standard deviations.

Knockout mutants of 93P10H1 (visit 10) and 93P12H1 (visit 12) were engineered using overlap extension PCR and homologous recombination as described previously (25). Three mutants were generated for each of the two isolates: *hmw1* knockouts, *hmw2* knockouts, and *hmw1/hmw2* double knockouts (Fig. 6A). Knocking out *hmw2* in isolate 93P10H1, which expressed HMW2A, results in a 2- to 3-log decrease in adherence to H292 human respiratory epithelial cells compared to the wild type, whereas knocking out *hmw1* in 93P12H1, which expressed HMW1A, results in a less prominent 1-log decrease in adherence (Fig. 6B). Knocking out *hmw1* in 93P12H1, which expressed HMW1A, results in a >1-log decrease in invasion compared to the wild type, whereas knocking out *hmw2* has no impact on invasion because it is not expressed in 93P12H1 (Fig. 6C). These results indicate that HMW2A functions primarily as an adhesin and that HMW1A functions as an adhesin and also facilitates invasion of human respiratory epithelial cells.

### Modulation of biofilm formation during persistence.

**FIG 7:**
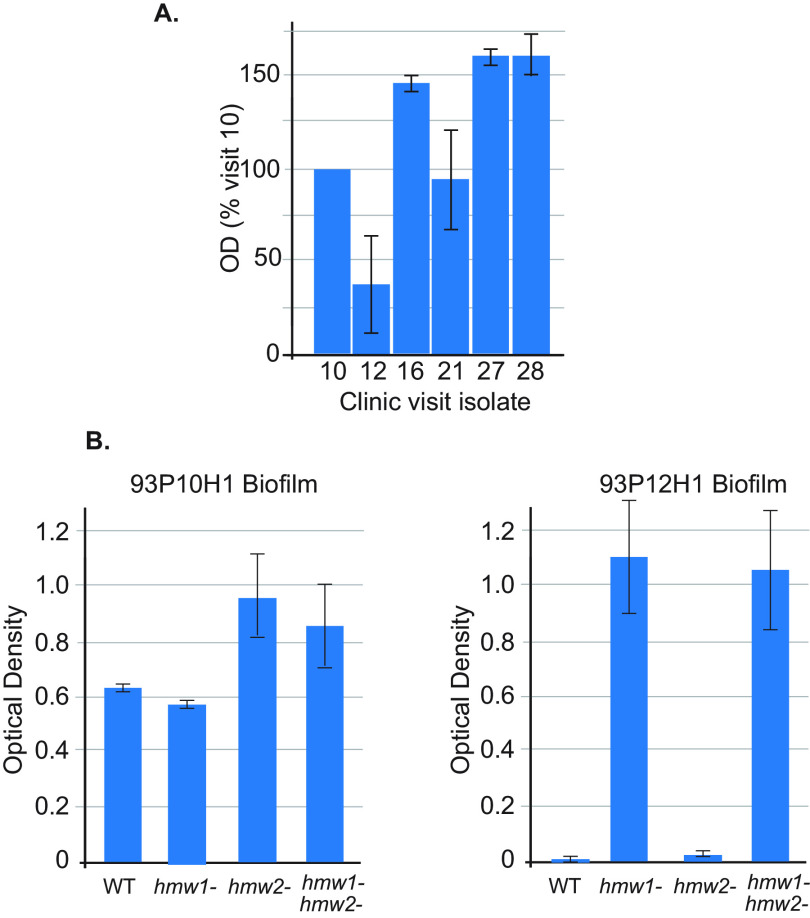
(A) Results of biofilm assays of selected isolates of the persistent NTHi strain, including the four isolates for which gap-free genome sequences are available in addition to selected isolates between visits 16 and 28 (392 days) as noted in the *x* axis. Results are expressed as a percentage of the visit 10 isolate calculated from the optical density at 570 nm (OD_570_). (B) Results of biofilm assays of isolates 93P10H1 (visit 10 isolate) and 93P12H1 (visit 12 isolate) and their corresponding knockout mutants as noted in the *x* axis. *y* axes show OD_570_, indicating level of biofilm formation. Values denoted by bars for all assays (A and B) represent the mean of three independent experiments. Error bars represent standard deviations.

To assess biofilm formation during persistence in COPD airways, serial isolates of the persistent strain were assayed for their capacity to form biofilms *in vitro*. Isolates that expressed HMW2A (visits 10, 16, 21) or neither HMW (visits 27 and 28) formed prominent biofilms, whereas the sole isolate that expressed HMW1A (visit 12) showed limited biofilm formation (Fig. 7A). HMW knockout mutants were assayed to determine the effect of HMWs on biofilm formation. In the case of isolate 93P10H1, which expresses HMW2A, knocking out either or both HMWs resulted in relatively minor changes in biofilm formation. By contrast, in the case of 93P12H1, which expresses HMW1A, knocking out expression of HMW1A resulted in a marked increase in biofilm formation (Fig. 7B). These results indicated that NTHi regulates biofilm formation during persistence in COPD airways and that biofilm formation is generally inversely related to HMW1 expression, consistent with the recent observations of Fernández-Calvet et al. (24).

## DISCUSSION

NTHi persists in the airways of adults with COPD for months to years, contributing to airway inflammation, increased symptoms, and progressive loss of pulmonary function that are hallmarks of the course of COPD (4, 6, 8, 13, 14). Little is known about the mechanisms by which NTHi persists in COPD airways. The present study advances our understanding of changes in the NTHi genome in human airways through detailed analysis of sequential isolates of a strain of NTHi that persisted in an adult with COPD, with a focus on genome rearrangements. This analysis revealed novel observations related to genome changes that modulate expression of key virulence molecules and phenotypes, advances previous work in elucidating mechanisms of persistence, and acts as a guide for future studies to understand bacterial persistence in human airways.

This study includes the following key observations: (i) a ~400-kb segment of the genome undergoes inversion several times during persistence in the human airways; (ii) the *hmw1* and *hmw2* operons are present at each end of the inversion and the inversion site is located in the promoter region of the *hmw* operons; (iii) NTHi modulates which HMW adhesin is expressed and the expression level of each HMW adhesin during persistence in COPD airways both by the ~400-kb inversion and by the number of 7-bp upstream repeats in the *hmw* promoter regions; (iv) HMW2A functions primarily as an adhesin and HMW1A functions as an adhesin and also facilitates invasion of human respiratory epithelial cells; and (v) biofilm formation is inversely correlated with HMW1A expression in human COPD airways.

We have previously shown in other strains that the ~400-kb segment of the NTHi genome undergoes inversion during persistence in two out of four persistent strains (15). This observation was based on analysis of the genomes of isolates recovered at the time of acquisition by the patient compared with the genome of the final isolate before clearance of the strain by the same patients as part of monthly sampling in a prospective study. The present work advances this observation by studying sequential isolates using gap-free genomes, revealing that the same ~400-kb segment inverts multiple times during persistence, suggesting a dynamic interplay between pathogen and host with regard to this genome rearrangement.

The genomes we analyzed are derived from single colonies of the pathogen isolated from sputum, raising the potential limitation that sampling of a single colony may not reflect the population of bacteria in the sputum. Analysis of multiple colonies collected from the original culture revealed that isolates from all individual colonies, at each of the four visits for which we have gap-free genomes, showed that the ~400-kb segment had the same orientation as in other isolates grown from colonies at that visit. In addition, we showed that the number of 7-bp repeats among isolates from individual colonies in a sputum sample paralleled those of the other isolates in the same sample. These observations are consistent with the conclusion that the entire population of NTHi in a sputum sample has the same orientation of the ~400-kb segment and similar numbers of 7-bp repeats in the promoter. We speculate that changing environmental conditions in the airway create a competitive advantage for one configuration, which replaces the other configuration, resulting in a dynamic cycling of the state of the strain in the airway.

The genes that encode HMW1A and HMW2A, which function as major adhesins of NTHi, are present in 40% to 80% of NTHi strains (26–30). Previous work by Cholon et al. (23) on longitudinally collected strains, which were part of the same prospective study as the strain described in this report (31), showed that expression of HMW1A and HMW2A decreased over time in a majority of patients, associated with increases in the number of 7-bp repeats. The present study confirms the association between HMW1A and HMW2A expression and the number of 7-bp repeats and advances our understanding with three novel observations. First, multiple inversions occurred during persistence, consistent with a dynamic host-pathogen interaction. Second, inversion of the ~400-kb segment of the genome during persistence in human airways appears to be an additional mechanism of regulation of HMW expression. And third, the expression of HMW2A during persistence underwent both up- and downregulation. The availability of four gap-free genomes was important in accurately delineating the inversion events that occurred during persistence. The prospective study design and examination of 14 sequential isolates of the persistent strain were critical in revealing that NTHi adapts rapidly to environmental fluctuations by modulating its genome through reversible rearrangements in the form of a large inversion.

The observation that the NTHi genome undergoes frequent inversion events of a ~400-kb segment during persistence in human airways suggests that the inversion event confers an advantage to persistence. Many bacterial genomes display a bias in which most genes are oriented in the direction of replication (32–35). However, inversions have been observed in several bacterial species and are often reversible and facilitate adaptation to specialized niches (36–38). We hypothesize that the inversion events which take place during NTHi persistence are adaptations triggered by continuously changing conditions in the COPD airway environment, facilitating persistence and thus contributing to virulence. We further speculate that the inversion event accelerates alterations in the number of repeats in the promoter, resulting in a balancing of the regulation of HMW expression in the airway. Replication is accompanied by more gradual changes in 7-bp repeats via slipped-strand mispairing, whereas an inversion event can alter repeats upstream of *hmw* genes more abruptly, immediately affecting which HMW is expressed. The relationship between inversion events and the expression of key virulence determinants—HMW1A and HMW2A—which mediate adherence, facilitate invasion, and act as a trigger for biofilm formation, further supports this hypothesis. Future work will explore the relationship between the level of adherence and invasion with the propensity of strains to elicit an inflammatory response in the epithelium.

HMW1A preferentially binds to the host cell glycan receptor, α2,3-linked *N*-sialyllactosamine (2,3-SLN), while HMW2A preferentially binds α2,6-linked *N*-sialyllactosamine (2,6-SLN) (21, 39). Interestingly, 2,3-SLN predominates in the lower airways, whereas 2,6-SLN is present throughout the human respiratory tract but more frequent in the upper airway (40). The observation that NTHi switches expression of HMW1A and HMW2A during persistence in COPD airways raises the intriguing speculation of whether differential expression of HMWs may trigger changes in clinical manifestations, including exacerbations, by mediating the movement of NTHi in the airway. For example, could a switch to HMW1A expression facilitate infection of the lower airways through higher-affinity binding to 2,3-SLN, which predominates in lower airways? Similarly, could a switch to HMW2A expression facilitate transmission of NTHi through binding 2,6-SLN, which predominates in upper airways? Aside from the initial exacerbation that occurred upon acquisition of the strain we studied, no changes in clinical status related to respiratory symptoms were detected during the persistence of the strain. Analysis of the relationship between inversion events, HMW expression, and clinical status during persistence in multiple strains will enable us to test these hypotheses.

Collectively, the observations related to HMW expression and biofilm formation are consistent with the concept that adhesin expression is critical for NTHi to initiate carriage in the airways. Once established, persistence favors downregulation of the adhesins to evade immune responses and activation of biofilm formation to survive in the airways (23, 24), The inverse correlation between HMW1 expression and biofilm formation was recently described in elegant work by Fernández-Calvet et al. (24). The present work advances that observation by elucidating the dynamics of the relationship between HMW1A expression and biofilm formation in sequential isolates of a strain that persisted for 543 days in a human COPD airway. We have not studied the underlying mechanism, but in working with our HMW knockout mutants, we observed differences in autoaggregation related to expression of HMW1A. This observation is consistent with the observation in the paper above that strains that express certain HMW1A alleles invade as aggregates while strains without HMW1A invade as single cells.

One limitation of this work is that it focuses on a single persistent strain. This targeted approach enabled us to perform a detailed analysis of 14 isolates recovered at different times during persistence and will inform subsequent studies of multiple strains using more efficient approaches. While NTHi show substantial genomic variability among strains, our previously reported analysis of genome rearrangements in four strains showed that two of the four underwent inversion of the same genome fragment some time during persistence in the airways and that the inversion sites were in or near the *hmw1* and *hmw2* operons (15). Therefore, the major rearrangements reported here are not unique to this strain. A second limitation noted above is that our genomes were determined from single colonies, raising the question of whether they accurately reflect the population of NTHi in the airway at the time of sampling. This limitation is partially addressed by analyzing NTHi grown from multiple individual colonies from the original culture plate. All 21 isolates from original colonies studied showed that the ~400-kb fragment was in the same orientation in all other isolates for each sputum sample. This approach may miss clones that represent less abundant populations in the sample but reassures us that we studied the predominant population in the airways at the time of sampling.

The present study advances our understanding of the mechanisms of persistence of NTHi in the airways of adults with COPD. The NTHi genome undergoes a genome rearrangement during persistence and this inversion impacts the regulation of expression of key virulence molecules that mediate or facilitate major virulence phenotypes, including adherence to respiratory epithelial cells, invasion of epithelial cells, and biofilm formation. The inversion appears to occur frequently, having occurred three times in ~18 months of persistence. Future studies will focus on identifying the events or conditions in the airways that trigger this inversion, quantitative assessment of the frequency with which the inversion occurs among NTHi strains, and approaches to exploit these observations to develop interventions which can eradicate or prevent chronic NTHi infection of airways in COPD.

## MATERIALS AND METHODS

The study was approved by the institutional review boards of the University at Buffalo and the Western New York Veterans Affairs Healthcare System. Written, informed consent was provided by participants.

### COPD study clinic.

The present study is based on one participant in a 20-year prospective observational study of adults with COPD that was conducted at the Buffalo Veterans Affairs Medical Center from 1994 to 2014 (10, 15, 31). Participants were seen at monthly clinic visits and at unscheduled visits at the onset of suspected acute exacerbations. Detailed clinical data, expectorated sputum samples, and serum samples were collected at each visit.

### Bacterial strains.

NTHi were identified using standard microbiology techniques. In addition, immunoblot assays of whole-cell lysates using a surface protein P6-specific monoclonal antibody were performed on each strain to distinguish NTHi from Haemophilus haemolyticus (41). Strains which were isolated at a single monthly clinic visit and not isolated again at subsequent visits were defined as cleared strains. Strains which were isolated from a study participant at sequential monthly clinic visit and were the same strain by MLST were defined as persistent strains. Previous work demonstrated that clinic visits which yielded negative cultures for NTHi but were preceded and followed by NTHi of the same sequence type (ST) represented persistence of that strain despite the negative culture (10). To determine MLSTs of isolates 93P10H1, 93P12H1, 93P16H1 and 93P28H1, reads mapping to the seven MLST genes were extracted from genomes and submitted to the MLST database (mlst.net) and a sequence type was assigned to each strain as previously described (42, 43). MLSTs of the remaining intervening isolates from the patient 93 strain were determined by PCR of MLST loci and sequence determination.

### Genome sequencing, assembly and analysis.

We used previously described methods (15, 44). Briefly, genomic DNA was extracted from low-passage NTHi strains (three passages from the original isolation) using the Gram-negative bacteria protocol of the Qiagen DNeasy Blood & Tissue kit. Four serial isolates from patient 93 (93P10H1, 93P12H1, 93P16H1, 93P28H1) were subjected to Pacific Biosciences (PacBio) Sequel II 2.0 sequencing to obtain gap-free genomes. Genomes were assembled into single contigs (gap-free genomes) with CANU v1.8 software (45), aligned to the H. influenzae NTHi 86-028NP reference nucleotide sequence (GenBank accession no. CP000057.2) using NUCmer (46) and rotated to make the first nucleotide the same as 86-028NP. The four gap-free, trimmed and rotated genome sequences were subjected to the IGS annotation pipeline (47) and submitted to GenBank under accession numbers CP116490 (93P10H1), CP116397 (93P12H1), CP116396 (93P16H1), and CP116489 (93P28H1).

### Immunoblot assays.

Whole-cell isolates of NTHi strains were subjected to immunoblot assays and probed with primary guinea pig anti-HMW1A (gp85) provided by Joseph St. Geme.

### Quantitative reverse transcriptase PCR (qRT-PCR).

Previously described methods were used (48, 49). Briefly, thawed bacterial pellets were resuspended in 500 mL RNA Wiz (RiboPure RNA Purification kit for bacteria; Ambion, Life Technologies, Grand Island, NY). RNA was isolated by chloroform extraction (0.2× volume; 100 μL) and centrifuged for 5 min at 4°C at 14,000 × *g*. The top aqueous layer (~200 μL) was mixed with 0.5× 100% ethanol (100 μL), further purified with RiboPure spin columns according to the manufacturer’s instructions, and eluted with 40 μL of elution solution (Ribopure kit) heated to 95°C. Residual DNA contamination was eliminated from 10 μg of RNA with the Promega DNase kit according to the manufacturer’s instructions, further purified with a Qiagen RNA Minikit according to the manufacturer’s instructions, and eluted with 40 μL of H_2_O. RNA was quantitated with a NanoDrop instrument (Thermo Fisher Scientific, Wilmington, DE) and frozen at −80°C in 1-μg aliquots until use. Integrity was assessed by electrophoresis through a 2% agarose denaturing morpholinepropanesulfonic acid (MOPS) gel. Primers were designed to amplify specific regions of *hmw1A*, *hmw2A*, and *ompp6*, which is constitutively expressed. Results were normalized to *ompp6*.

### Adherence and invasion assays.

Twenty-four-well plates were seeded with suspensions of the H292 human respiratory epithelial cell line (2 × 10^5^ cells per well) and grown using previously described methods (49). Adherence and invasion assays were performed in duplicate wells. NTHi was grown overnight on chocolate agar. Colonies were suspended in broth to an optical density at 600 nm (OD_600_) of 0.08. This suspension was used to inoculate broth cultures, which were grown to mid-log phase (OD_600_ of ~0.4) with shaking. H292 cells were washed twice with fresh RPMI medium, inoculated with NTHi at a multiplicity of infection (MOI) of 1 bacterium per H292 cell, and the plates were centrifuged at 170 × *g* for 5 min at room temperature to facilitate contact with bacteria. Cells and bacteria were incubated for 3 h at 37°C with 5% CO_2_. Nonadherent cells were removed by gently washing the wells 3 times with phosphate-buffered saline. To quantify adherent cells, 200 μL of trypsin (0.25%) was added to each well and plates were incubated at 37°C for 10 min to remove adherent cells. A 300-μL volume of 1% saponin was added to each well and the contents were pipetted into microfuge tubes and, after vigorous vortexing, plated in duplicate to determine bacterial cell counts. Adherence was measured as CFU/mL.

To assess intracellular invasion of NTHi in the epithelial cells, infected monolayers were washed 3 times with phosphate-buffered saline and RPMI containing 50 μg/mL gentamicin and incubated for 1 h at 37°C. After washing, 200 μL trypsin (0.25%) was added to each well, and plates were incubated at 37°C for 10 min to remove adherent cells. A 300-μL volume of 1% saponin was applied to each well to lyse cells and pipetted into microcentrifuge tubes, and, after vigorous vortexing, the contents were plated in duplicate to perform bacterial cell counts. Invasion was measured as CFU/mL. Each experiment was repeated 3 times and the mean and standard deviation were calculated. Statistical significance was determined by performing a two-tailed *t* test. A *P* of <0.05 was considered significant.

### Biofilm assays.

Previously described methods were used (50, 51). Briefly, an overnight NTHi broth culture was diluted 1:200 in fresh broth and 200 μL was inoculated into the wells of a 96-well CELLSTAR tissue culture plate (Greiner Bio-One, Monroe, NC). The plates were incubated at 37°C on a nutator for 24 h. Before biofilm quantitation, growth was assessed by measuring the OD_490_ in a Bio-Rad plate reader. To quantitate biofilm formation, 20 μL of Difco crystal violet (Becton, Dickinson and Co., Sparks, MD) was added to each well and incubated at room temperature for 15 min. Wells were washed vigorously with distilled water and the plate was air-dried. A 230-μL volume of 95% ethanol was added to each well and the OD_570_ was measured. Each plate included four control wells which contained sterile broth instead of bacteria but were otherwise treated identically. The OD_570_ was standardized against these wells. Strains were tested in quadruplicate wells. The mean and standard deviation were calculated from values for each strain. Comparison of biofilm formation between mutant and parent strains was performed using an unpaired *t* test.
