# Supplementary material for: Adaptation of Nontypeable Haemophilus influenzae in Human Airways in COPD: Genome Rearrangements and Modulation of Expression of HMW1 and HMW2
Source: mBio. 2023 Mar 16;14(2):e00140-23. doi: 10.1128/mbio.00140-23 (PMC10127715; doi:10.1128/mbio.00140-23)
Supplement: TABLE S1 [file mbio.00140-23-s0001.docx]

**Table S1.** Results of quantitative reverse transcriptase PCR of Patient 93 isolates*^a^* grown in vitro

| **Isolate** | ***hmw1* transcription replicates** | ***hmw1***  **Mean** | ***hmw1***  **standard deviation** | ***hmw2* transcription replicates** | ***hmw2***  **Mean** | ***hmw2***  **standard deviation** |
| --- | --- | --- | --- | --- | --- | --- |
| 93P10H1 | \| 0.0041 \| \| --- \| \| 0.0036 \| \| 0.0065 \| | 0.0047 | 0.0012 | \| 0.0924 \| \| --- \| \| 0.1457 \| \| 0.1437 \| | 0.1272 | 0.0246 |
| 93P12H1 | \| 0.1912 \| \| --- \| \| 0.2066 \| \| 0.1507 \| | 0.1828 | 0.0235 | \| 0.0035 \| \| --- \| \| 0.0070 \| \| 0.0070 \| | 0.0059 | 0.0016 |
| 93P16H1 | \| 0.0107 \| \| --- \| \| 0.0041 \| \| 0.0102 \| | 0.0083 | 0.0030 | \| 0.0670 \| \| --- \| \| 0.0431 \| \| 0.0622 \| | 0.0574 | 0.0103 |
| 93P21H1 | \| 0.0082 \| \| --- \| \| 0.0176 \| \| 0.0186 \| | 0.0148 | 0.0047 | \| 0.1717 \| \| --- \| \| 0.1825 \| \| 0.1444 \| | 0.1662 | 0.0160 |
| 93P23H1 | \| 0.0162 \| \| --- \| \| 0.0221 \| \| 0.0042 \| | 0.0142 | 0.0074 | \| 0.0245 \| \| --- \| \| 0.0246 \| \| 0.0060 \| | 0.0184 | 0.0087 |
| 93P27H1 | \| 0.0013 \| \| --- \| \| 0.0021 \| \| 0.0091 \| | 0.0042 | 0.0034 | \| 0.0066 \| \| --- \| \| 0.0037 \| \| 0.0111 \| | 0.0071 | 0.0030 |
| 93P28H1 | \| 0.0060 \| \| --- \| \| 0.0017 \| \| 0.0031 \| | 0.0036 | 0.0018 | \| 0.0094 \| \| --- \| \| 0.0058 \| \| 0.0051 \| | 0.0068 | 0.0018 |

*^a^*Isolates studied by qRT-PCR include those on which gap-free genome sequences were determined in addition to selected isolates in the interval between visits 16 and 28 (392 days).
