# Supplementary material for: Adaptation of Nontypeable Haemophilus influenzae in Human Airways in COPD: Genome Rearrangements and Modulation of Expression of HMW1 and HMW2
Source: mBio. 2023 Mar 16;14(2):e00140-23. doi: 10.1128/mbio.00140-23 (PMC10127715; doi:10.1128/mbio.00140-23)
Supplement: TABLE S2 [file mbio.00140-23-s0002.docx]

**Table S2.** Number of repeats in the *hmw* promoters in isolates grown from individual colonies from the original sputum culture plates of the four isolates on which gap-free genomic sequences are available.

| Isolate Colonies | HMW1  7 bp repeats | HMW2  7 bp repeats |
| --- | --- | --- |
| 93P10H1 | 53 | 14 |
| 93P10H2 | 58 | 13 |
| 93P10H3 | 60 | 12 |
| 93P10H5 | 56 | 13 |
| 93P10H6 | 57 | 13 |
| 93P10H8 | 58 | 14 |
| 93P12H1 | 11 | 55 |
| 93P12H2 | 11 | 49 |
| 93P16H1 | 47 | 16 |
| 93P16H2 | 56 | 19 |
| 93P16H3 | 56 | 18 |
| 93P16H4 | 56 | 19 |
| 93P28H1 | 43 | 63 |
| 93P28H2 | 52 | 54 |
| 93P28H3 | 44 | 55 |
| 93P28H4 | 36 | 53 |
| 93P28H5 | 49 | 53 |
| 93P28H6 | 34 | 55 |
| 93P28H7 | 46 | 57 |
| 93P28H8 | 51 | 52 |
| 93P28H9 | 44 | 58 |
